# Supplementary material for: Long-Term Functional Hyperemia after Uncomplicated Phacoemulsification: Benefits beyond Restoring Vision
Source: Diagnostics (Basel). 2022 Oct 10;12(10):2449. doi: 10.3390/diagnostics12102449 (PMC9600879; doi:10.3390/diagnostics12102449)
Supplement: Supplementary file 1 [file diagnostics-12-02449-s001.zip › diagnostics-1957387-supplementary.pdf]

Article Title: Long-term functional hyperemia after uncomplicated phacoemulsification: benefits beyond restoring vision

Journal Name: Diagnostics

Author Names: Ana Ćurić, Mirjana Bjeloš, Mladen Bušić, Biljana Kuzmanović Elabjer, Benedict Rak, Nenad Vukojević

Corresponding author:

Mirjana Bjeloš

University Eye Department, University Hospital "Sveti Duh"

Sveti Duh 64, Zagreb, Croatia

e-mail: dr.mbjelos@gmail.com

This article contains additional Supplementary Material: Supplementary Tables S1-S9.

Table S1. Statistical analysis of changes in vascular parameters in nerve fiber layer vascular plexus.

NFLVP	Before	1 week after	1 month after	3 months after	6 months after	*p*	Change
EA (mm^2)	8.3795 (8.3770-8.3804)	8.3796 (8.3770-8.3805)	8.3794 (8.3768-8.3807)	8.3794 (8.3768-8.3807)	8.3794 (8.3768-8.3803)	0.889	0.01%
**VA (mm^2)**	1.8591 (1.4153-2.3589)	2.5353 (2.0353-3.0919)	2.6530 (2.1733-3.2279)	2.7846 (2.2839-3.1992)	2.8117 (2.3376-3.2735)	**<0.001**	**36.37%**
**VPA (%)**	22.1838 (16.8914-28.1471)	30.2547 (24.2985-36.8916)	31.6644 (25.9395-38.5160)	33.2328 (27.2585-38.1763)	33.5613 (27.8909-39.1569)	**<0.001**	**36.38%**
**TNJ**	495 (320-731)	809 (593-1049)	862 (644-1109)	906 (686-1111)	936 (728-1140)	**<0.001**	**63.43%**
**JD (junctions/mm^2)**	59.2552 (38.1612-87.2170)	96.5340 (70.7350-125.1407)	102.8470 (76.8021-132.3245)	108.1508 (81.8590-132.6084)	111.6791 (87.0387-136.0834)	**<0.001**	**62.91%**
**TVL (mm)**	71.1006 (53.6238-92.1387)	95.0071 (77.8507-112.2952)	101.7714 (83.9617-118.3007)	104.4058 (85.9790-118.2746)	103.9301 (87.8332-118.7705)	**<0.001**	**33.62%**
**AVL (mm)**	0.1716 (0.1297-0.2246)	0.2620 (0.1758-0.3935)	0.2712 (0.2059-0.4137)	0.3000 (0.2071-0.4194)	0.2973 (0.2156-0.4074)	**<0.001**	**52.68%**
TNEP	999 (855-1140)	1021 (882-1135)	998 (864-1106)	994 (858-1110)	975 (891-1107)	0.369	2.20%
**ML**	0.3649 (0.2617-0.5047)	0.2294 (0.1778-0.3309)	0.2098 (0.1653-0.2757)	0.2106 (0.1569-0.2718)	0.2053 (0.1588-0.2881)	**<0.001**	**-37.12%**

NFLVP = nerve fiber layer vascular plexus; EA = explant area; VA = vessels area; VPA = vessels percentage area; TNJ = total number of junctions; JD = junctions density; TVL = total vessels length; AVL = average vessels length; TNEP = total number of end points; ML = mean lacunarity.

This table shows median and interquartile ranges for each parameter (25th and 75th percentile). *P* values and percentages of change are presented one week after phacoemulsification.

Friedman ANOVA test, significant difference (bold values) was found for values with $p < 0.001$.

Table S2. Statistical analysis of changes in vascular parameters in superficial vascular plexus.

SVP	Before	1 week after	1 month after	3 months after	6 months after	*p*	Change
EA (mm^2)	8.3801 (8.3790-8.3806)	8.3801 (8.3790-8.3806)	8.3801 (8.3793-8.3807)	8.3801 (8.3790-8.3809)	8.3801 (8.3790-8.3807)	0.125	0.00%
**VA (mm^2)**	4.5545 (4.0094-4.9355)	5.0687 (4.8105-5.3193)	5.0724 (4.8006-5.3900)	5.1967 (4.9060-5.4451)	5.1283 (4.9467-5.4279)	**<0.001**	**11.29%**
**VPA (%)**	54.5669 (47.8463-58.8991)	60.4780 (57.4106-63.5375)	60.5694 (57.7880-64.3410)	62.0127 (58.5389-64.9757)	61.2477 (59.0638-64.7752)	**<0.001**	**10.83%**
**TNJ**	1314 (1115-1397)	1432 (1373-1485)	1441 (1366-1502)	1446 (1383-1507)	1439 (1381-1496)	**<0.001**	**8.98%**
**JD (junctions/ mm^2)**	156.8099 (133.0305-166.6892)	170.9058 (163.7611-177.2097)	171.9498 (163.3842-179.8371)	172.7219 (165.0255-179.8147)	172.6123 (165.0242-178.5276)	**<0.001**	**8.99%**
**TVL (mm)**	138.455 (127.844-144.633)	146.063 (142.680-149.708)	147.222 (142.383-151.002)	147.244 (144.288-150.959)	146.856 (143.387-150.129)	**<0.001**	**5.49%**
**AVL (mm)**	3.957 (1.790-6.092)	6.059 (4.628-8.532)	6.120 (4.816-9.000)	6.559 (4.915-8.759)	6.135 (4.760-8.243)	**<0.001**	**53.13%**
**TNEP**	240 (194-412)	169 (147-210)	170 (147-206)	168 (140-197)	170 (146-199)	**<0.001**	**-29.58%**
**ML**	0.0504 (0.0407-0.072)	0.0398 (0.0322-0.0474)	0.0378 (0.0307-0.0457)	0.0377 (0.0311-0.0456)	0.0385 (0.0315-0.0469)	**<0.001**	**-20.97%**

SVP = superficial vascular plexus; EA = explant area; VA = vessels area; VPA = vessels percentage area; TNJ = total number of junctions; JD = junctions density; TVL = total vessels length; AVL = average vessels length; TNEP = total number of end points; ML = mean lacunarity. This table shows median and interquartile ranges for each parameter (25th and 75th percentile). *P* values and percentages of change are presented one week after phacoemulsification. Friedman ANOVA test, significant difference (bold values) was found for values with $p < 0.001$.

Table S3. Statistical analysis of changes in vascular parameters in superficial vascular complex.

SVC	Before	1 week after	1 month after	3 months after	6 months after	*p*	Change
EA (mm^2)	8.3799 (8.3787-8.3806)	8.3799 (8.3787-8.3806)	8.3801 (8.3794-8.3807)	8.3799 (8.3787-8.3807)	8.3799 (8.3787-8.3807)	0.889	0.01%
**VA (mm^2)**	3.864 (3.127-4.329)	4.637 (4.297 to 4.918)	4.703 (4.311-5.021)	4.831 (4.453-5.007)	4.748 (4.451-4.989)	**<0.001**	**20.00%**
**VPA (%)**	46.103 (37.310-51.652)	55.323 (51.302-58.753)	56.133 (51.491-59.923)	57.639 (53.137-59.854)	56.710 (53.113-59.929)	**<0.001**	**20.00%**
**TNJ**	1143 (895-1306)	1384 (1286-1475)	1394 (1285-1487)	1424 (1327-1479)	1406 (1320-1476)	**<0.001**	**21.08%**
**JD (junctions/ mm^2)**	137.096 (106.731-155.833)	165.132 (153.665-176)	166.338 (153.287-177.447)	170.020 (158.352-176.456)	167.989 (157.466-177.210)	**<0.001**	**20.45%**
**TVL (mm)**	128.843 (110.867-138.255)	143.106 (137.009-147.567)	143.976 (137.635-148.805)	145.559 (140.618-149.084)	144.972 (140.884-148.726)	**<0.001**	**11.07%**
**AVL (mm)**	2.028 (0.760-3.547)	5.389 (3.687-7.106)	5.292 (3.600-7.417)	5.996 (4.079-8.904)	5.454 (4.148-8.194)	**<0.001**	**165.76%**
**TNEP**	406 (285-614)	224 (184-278)	208 (180-276)	201 (166-271)	204 (176-250)	**<0.001**	**-44.83%**
**ML**	0.0727 (0.0523-0.115)	0.0487 (0.0403-0.0588)	0.0481 (0.0385-0.0562)	0.0458 (0.0377-0.0564)	0.0475 (0.0392-0.0585)	**<0.001**	**-33.06%**

SVC = superficial vascular complex; EA = explant area; VA = vessels area; VPA = vessels percentage area; TNJ = total number of junctions; JD = junctions density; TVL = total vessels length; AVL = average vessels length; TNEP = total number of end points; ML = mean lacunarity.
This table shows median and interquartile ranges for each parameter (25th and 75th percentile). *P* values and percentages of change are presented one week after phacoemulsification.
Friedman ANOVA test, significant difference (bold values) was found for values with $p < 0.001$.

Table S4. Statistical analysis of changes in vascular parameters in intermediate capillary plexus.

ICP	Before	1 week after	1 month after	3 months after	6 months after	*p*	Change
EA (mm²)	8.3800 (8.3774-8.3806)	8.3797 (8.3779-8.3808)	8.3801 (8.3788-8.3809)	8.3800 (8.3786-8.3808)	8.3800 (8.3786-8.3808)	0.755	0.00%
**VA (mm²)**	4.4589 (3.6848-4.7876)	5.0306 (4.7727-5.2698)	5.0145 (4.7338-5.2741)	5.1236 (4.7383-5.3024)	5.0714 (4.8539-5.2669)	**<0.001**	**12.82%**
**VPA (%)**	53.2082 (53.2082-57.1352)	60.0233 (56.9550-62.8829)	59.8439 (56.4824-62.9344)	61.1325 (56.5415-63.2928)	60.5677 (57.9167-62.8452)	**<0.001**	**12.81%**
**TNJ**	1436 (1121-1559)	1604 (1546-1693)	1617 (1536-1713)	1632 (1555-1692)	1647 (1573-1693)	**<0.001**	**11.70%**
**JD (junctions/mm²)**	171.3555 (133.7933-185.9862)	191.3835 (184.4353-201.9707)	192.9544 (183.2330-204.3767)	194.7350 (185.5743-201.8687)	196.5190 (187.7151-202.3389)	**<0.001**	**11.69%**
**TVL (mm)**	146.6273 (126.3234-152.6902)	154.7071 (152.0413-159.6729)	156.4076 (151.4608-160.6250)	156.7169 (152.8764-159.7203)	156.6050 (154.2605-159.6352)	**<0.001**	**5.51%**
**AVL (mm)**	3.1479 (1.1489-4.3315)	6.0974 (4.1989-8.2731)	5.4723 (3.7972-7.0673)	5.6966 (4.0042-7.7599)	5.8670 (4.1286-7.0363)	**<0.001**	**93.70%**
**TNEP**	340 (263-540)	212 (184-250)	225 (184-250)	208 (176-258)	211 (186-248)	**<0.001**	**-37.65%**
**ML**	0.03600 (0.02781-0.06323)	0.02584 (0.02011-0.03056)	0.02427 (0.01990-0.02934)	0.02442 (0.02016-0.02882)	0.02448 (0.01952-0.02880)	**<0.001**	**-28.22%**

ICP = intermediate capillary plexus; EA = explant area; VA = vessels area; VPA = vessels percentage area; TNJ = total number of junctions; JD = junctions density; TVL = total vessels length; AVL = average vessels length; TNEP = total number of end points; ML = mean lacunarity.

This table shows median and interquartile ranges for each parameter (25th and 75th percentile). *P* values and percentages of change are presented one week after phacoemulsification.

Friedman ANOVA test, significant difference (bold values) was found for values with $p < 0.001$.

Table S5. Statistical analysis of changes in vascular parameters in deep capillary plexus.

DCP	Before	1 week after	1 month after	3 months after	6 months after	*p*	Change
EA (mm^2)	8.3801 (8.3766-8.3803)	8.3802 (8.3790-8.3808)	8.3802 (8.3789-8.3807)	8.3801 (8.3790-8.3810)	8.3801 (8.3790-8.3810)	0.785	0.01%
**VA (mm^2)**	4.3672 (3.8253-4.7674)	4.9242 (4.6452-5.2837)	4.9290 (4.7928-5.3246)	5.0187 (4.7728-5.2834)	5.0594 (4.7865-5.2373)	**<0.001**	**12.75%**
**VPA (%)**	52.1056 (45.6493-56.8821)	58.7513 (55.4292-63.0430)	58.8133 (57.1941-63.5351)	59.9478 (57.4191-63.0429)	60.4815 (57.1285-62.6035)	**<0.001**	**12.75%**
**TNJ**	1385 (1220-1554)	1548 (1479-1656)	1602 (1508-1655)	1595 (1531-1649)	1596 (1511-1666)	**<0.001**	**11.77%**
**JD (junctions/ mm^2)**	165.2630 (145.6565-185.4727)	184.7808 (176.4101-197.5626)	191.4179 (179.9524-197.4751)	190.3027 (182.6724-197.1309)	190.4480 (180.2703-199.2827)	**<0.001**	**11.81%**
**TVL (mm)**	139.7239 (128.3934-149.5305)	150.9558 (146.4668-156.3002)	153.2497 (148.1792-156.9497)	152.7475 (148.6371-157.0685)	152.9109 (147.9636-156.4881)	**<0.001**	**8.04%**
**AVL (mm)**	2.4405 (1.3330-4.3567)	4.6059 (3.3411-6.7264)	5.6519 (3.9798-7.1728)	5.5208 (3.9462-6.9299)	5.4975 (3.9042-6.9947)	**<0.001**	**88.72%**
**TNEP**	360 (252-511)	238 (179-284)	216 (175-271)	214 (179-257)	212 (185-251)	**<0.001**	**-33.89%**
**ML**	0.06161 (0.04602-0.08830)	0.04423 (0.03464-0.05317)	0.04033 (0.03203-0.04841)	0.04050 (0.03270-0.04964)	0.04157 (0.03396-0.05088)	**<0.001**	**-28.21%**

DCP = deep capillary plexus; EA = explant area; VA = vessels area; VPA = vessels percentage area; TNJ = total number of junctions; JD = junctions density; TVL = total vessels length; AVL = average vessels length; TNEP = total number of end points; ML = mean lacunarity.

This table shows median and interquartile ranges for each parameter (25th and 75th percentile). *P* values and percentages of change are presented one week after phacoemulsification.

Friedman ANOVA test, significant difference (bold values) was found for values with $p < 0.001$.

Table S6. Statistical analysis of changes in vascular parameters in deep vascular complex.

DVC	Before	1 week after	1 month after	3 months after	6 months after	*p*	Change
EA (mm²)	8.3799 (8.3791-8.3807)	8.3799 (8.3788-8.3808)	8.3800 (8.3791-8.3810)	8.3799 (8.3788-8.3808)	8.3799 (8.3788-8.3808)	0.128	0.01%
**VA (mm²)**	4.7510 (4.0670-5.1796)	5.3422 (5.1466-5.6861)	5.4149 (5.2065-5.6675)	5.5216 (5.2554-5.6775)	5.5135 (5.2763-5.6330)	**<0.001**	**12.44%**
**VPA (%)**	56.6918 (48.5284-61.8060)	63.7460 (61.4178-67.8484)	64.6211 (62.1213-67.6476)	65.8857 (62.7092-67.7643)	65.7903 (62.9728-67.2612)	**<0.001**	**12.44%**
**TNJ**	1608 (1331-1707)	1727 (1674-1773)	1752 (1687-1786)	1738 (1697-1786)	1738 (1688-1779)	**<0.001**	**7.40%**
**JD (junctions/ mm²)**	191.8826 (159.0030-203.6972)	206.0519 (199.7749-211.4806)	209.1735 (201.2836-213.1473)	207.4106 (202.9628-213.0718)	207.4106 (201.5647-212.2858)	**<0.001**	**7.38%**
**TVL (mm)**	153.2259 (134.9415-159.5338)	160.7572 (157.9655-163.2968)	161.7421 (158.5118-163.9697)	161.7186 (158.4459-163.5630)	160.7295 (158.9470-163.2466)	**<0.001**	**4.92%**
**AVL (mm)**	4.8106 (1.3235-10.6267)	13.7022 (8.7047-21.8198)	13.5446 (9.3401-20.2722)	16.3397 (9.1956-22.6235)	14.4311 (9.8113-18.5240)	**<0.001**	**184.83%**
**TNEP**	272 (177-479)	142 (105-176)	133 (109-162)	119 (101-160)	124 (103-157)	**<0.001**	**-47.79%**
**ML**	0.03666 (0.02911-0.06960)	0.02667 (0.02151-0.03206)	0.02542 (0.02017-0.03085)	0.02482 (0.02054-0.02973)	0.02674 (0.02075-0.03134)	**<0.001**	**-27.24%**

DVC = deep vascular complex; EA = explant area; VA = vessels area; VPA = vessels percentage area; TNJ = total number of junctions; JD = junctions density; TVL = total vessels length; AVL = average vessels length; TNEP = total number of end points; ML = mean lacunarity.

This table shows median and interquartile ranges for each parameter (25th and 75th percentile). *P* values and percentages of change are presented one week after phacoemulsification.

Friedman ANOVA test, significant difference (bold values) was found for values with $p < 0.001$.

Table S7. Statistical analysis of changes in vascular parameters in retina.

RETINA	Before	1 week after	1 month after	3 months after	6 months after	*p*	Change
EA (mm^2)	8.3796 (8.3782-8.3805)	8.3796 (8.3781-8.3807)	8.3798 (8.3789-8.3805)	8.3800 (8.3794-8.3808)	8.3800 (8.3794-8.3808)	0.567	0.01%
**VA (mm^2)**	4.4098 (3.9080-4.8648)	4.9427 (4.7097-5.1777)	5.0057 (4.7243-5.2723)	5.1038 (4.8357-5.3189)	5.0444 (4.8364-5.3010)	**<0.001**	**12.09%**
**VPA (%)**	52.6185 (46.6445-58.0554)	58.9799 (56.1958-61.7856)	59.7317 (56.3762-62.9090)	60.9041 (57.7023-63.4796)	60.1941 (57.7098-63.2794)	**<0.001**	**12.09%**
**TNJ**	1529 (1348-1683)	1709 (1637-1771)	1730 (1654-1788)	1746 (1670-1787)	1734 (1670-1787)	**<0.001**	**11.77%**
**JD (junctions/ mm^2)**	182.4319 (160.8864-200.7545)	204.0459 (195.3320-211.3452)	206.4779 (197.4950-213.3626)	208.4055 (199.2211-213.2483)	207.3842 (199.2396-213.5274)	**<0.001**	**11.85%**
**TVL (mm)**	150.7949 (139.6191-158.1094)	159.9335 (155.9889-162.6339)	160.7585 (156.4810-164.0373)	161.6095 (156.9008-163.6342)	160.9889 (156.9437-162.8714)	**<0.001**	**6.06%**
**AVL (mm)**	2.5953 (1.2544-4.8658)	7.4237 (4.1864-10.6714)	7.7464 (4.5762-11.6792)	7.1950 (4.9531-10.3802)	7.6356 (5.0180-11.6167)	**<0.001**	**186.04%**
**TNEP**	359 (265-560)	210 (184-303)	207 (159-270)	192 (154-247)	192 (161-256)	**<0.001**	**-41.50%**
**ML**	0.03956 (0.03072-0.06329)	0.02937 (0.02439-0.03750)	0.02904 (0.02317-0.03310)	0.02678 (0.02315-0.03221)	0.02853 (0.02292-0.03490)	**<0.001**	**-25.76%**

EA = explant area; VA = vessels area; VPA = vessels percentage area; TNJ = total number of junctions; JD = junctions density; TVL = total vessels length; AVL = average vessels length; TNEP = total number of end points; ML = mean lacunarity.This table shows median and interquartile ranges for each parameter (25th and 75th percentile). *P* values and percentages of change are presented one week after phacoemulsification.
Friedman ANOVA test, significant difference (bold values) was found for values with $p < 0.001$.

Table S8. Statistical analysis of changes in vascular parameters in choriocapillaris.

CC	Before	1 week after	1 months after	3 months after	6 months after	*p*	Change
EA (mm^2)	8.3802 (8.3792-8.3808)	8.3800 (8.3791-8.3807)	8.3802 (8.3792-8.3808)	8.3806 (8.3794-8.3810)	8.3806 (8.3794-8.3810)	0.742	0.00%
VA (mm^2)	6.4122 (6.1682-6.5987)	6.4489 (6.2490-6.6267)	6.4555 (6.3336-6.6340)	6.4811 (6.3198-6.6653)	6.5013 (6.3096-6.6464)	0.279	0.57%
VPA (%)	76.5091 (73.6006-78.7315)	76.9443 (74.6011-79.0804)	77.0477 (75.5775-79.1660)	77.4137 (75.4058-79.5406)	77.6670 (75.2858-79.3051)	0.243	0.57%
TNJ	1721 (1597-1666)	1713 (1673-1760)	1707 (1673-1756)	1707 (1665-1744)	1705 (1658-1757)	0.834	-0.46%
JD (junctions/mm²)	205.3361 (198.7315-210.2192)	204.3816 (199.6236-209.9921)	203.7769 (199.6878-209.5172)	203.6657 (198.6613-208.0507)	203.4641 (197.8568-209.6402)	0.789	-0.46%
TVL (mm)	163.5641 (161.1857-164.8765)	163.4505 (161.5202-164.9303)	163.2078 (161.3249-164.8354)	163.1552 (161.4501-164.5270)	163.1552 (161.0359-165.2222)	0.914	-0.07%
AVL (mm)	39.7701 (24.5450-82.0256)	53.9182 (32.4966-81.4227)	54.6377 (39.5678-138.3566)	54.4731 (33.0892-157.7326)	55.1272 (40.6070-81.4441)	0.008	35.57%
**TNEP**	86 (65-116)	77 (57-98)	74 (58-91)	72 (54-96)	73 (57-87)	**<0.001**	**-10.47%**
ML	0.007167 (0.006056-0.009225)	0.006810 (0.005702-0.008304)	0.006560 (0.005650-0.007455)	0.006474 (0.005571-0.007438)	0.006331 (0.005625-0.007387)	0.001	-4.99%

CC = choriocapillaris; EA = explant area; VA = vessels area; VPA = vessels percentage area; TNJ = total number of junctions; JD = junctions density; TVL = total vessels length; AVL = average vessels length; TNEP = total number of end points; ML = mean lacunarity. This table shows median and interquartile ranges for each parameter (25th and 75th percentile). *P* values and percentages of change are presented for values one week after phacoemulsification. Friedman ANOVA test, significant difference (bold values) was found for values with $p < 0.001$.

Table S9. Statistical analysis of changes in vascular parameters in choroid.

CHOROID	Before	1 week after	1 month after	3 months after	6 months after	*p*	Change
EA (mm^2)	8.3802 (8.3791-8.3812)	8.3803 (8.3791-8.3812)	8.3800 (8.3791-8.3807)	8.3800 (8.3791-8.3809)	8.3801 (8.3791-8.3809)	0.359	0.00%
**VA (mm^2)**	5.6874 (5.4705-5.8991)	5.5657 (5.3586-5.7712)	5.5691 (5.3594-5.7349)	5.5739 (5.3481-5.7323)	5.5278 (5.3234-5.7335)	**<0.001**	**-2.14%**
**VPA (%)**	67.8640 (65.2963-70.3878)	66.4090 (63.9407-68.8593)	66.4687 (64.0577-68.4274)	66.5272 (63.9207-68.4411)	66.1934 (63.5885-68.4478)	**<0.001**	**-2.14%**
TNJ	1499 (1453-1564)	1530 (1469-1596)	1549 (1483-1589)	1537 (1492-1603)	1539 (1499-1590)	0.001	1.32%
**JD (junctions/ mm^2)**	180.5265 (173.5863-186.6140)	182.9515 (175.4667-190.4935)	184.8182 (176.9989-189.6404)	183.4202 (178.0604-191.2431)	183.7723 (178.8927-189.8698)	**<0.001**	**1.34%**
TVL (mm)	152.9726 (149.5086-155.7017)	153.0903 (149.4116-155.8366)	153.6118 (150.1565-156.1263)	153.0702 (150.7052-155.9868)	153.2636 (150.6808-155.9098)	0.319	0.08%
AVL (mm)	10.0777 (6.5411-15.3401)	10.1020 (6.4623-14.2535)	9.7952 (7.1834-15.4482)	11.5864 (7.0002-15.7957)	11.6441 (6.8459-15.8033)	0.839	0.24%
**TNEP**	199 (162-249)	215 (172-250)	208 (176-244)	210 (168-255)	206 (174-258)	**<0.001**	**8.04%**
ML	0.01311 (0.01130-0.01628)	0.01382 (0.01174-0.01658)	0.01383 (0.01176-0.01569)	0.01348 (0.01165-0.01671)	0.01373 (0.01164-0.01597)	0.113	5.44%

EA = explant area; VA = vessels area; VPA = vessels percentage area; TNJ = total number of junctions; JD = junctions density; TVL = total vessels length; AVL = average vessels length; TNEP = total number of end points; ML = mean lacunarity.

The table shows median and interquartile ranges for each parameter (25th and 75th percentile). *P* values and percentages of change are presented one week after phacoemulsification.

Friedman ANOVA test, significant difference (bold values) was found for values with $p < 0.001$.
